# How education and GDP drive the COVID-19 vaccination campaign

**DOI:** 10.1186/s13690-022-00924-0

**Published:** 2022-07-18

**Authors:** Vu M. Ngo, Klaus F. Zimmermann, Phuc V. Nguyen, Toan L. D. Huynh, Huan H. Nguyen

**Affiliations:** 1 University of Economics Ho Chi Minh City, Ho Chi Minh City, Vietnam; 2 Global Labor Organization (GLO), Essen, Germany; 3 UNU-MERIT & Maastricht University, Maastricht, Netherlands; 4 Centre for Economic Policy Research (CEPR), London, UK; 5 Massey University, Palmerston North, New Zealand

**Keywords:** COVID-19, Educational development, Political regimes, Economic strength, Vaccination

## Abstract

**Background:**

Since vaccination is the decisive factor for controlling the COVID-19 pandemic, it is important to understand the process of vaccination success which is not well understood on a global level. The study is the first to judge the now completed "first wave" of the vaccination efforts. The analysis is very relevant for the understanding why and where the vaccination process observed got stuck by the end of 2021.

**Methods:**

Using data from 118 countries globally and weighted least squared and survival analysis, we identify a variety of factors playing crucial roles, including the availability of vaccines, pandemic pressures, economic strength measured by Gross Domestic Product (GDP), educational development, and political regimes.

**Results:**

Examining the speed of vaccinations across countries until the Fall of 2021 when the global process got stuck, we find that initially authoritarian countries are slow in the vaccination process, while education is most relevant for scaling up the campaign, and the economic strength of the economies drives them to higher vaccination rates. In comparison to North and Middle America, European and Asian countries vaccinated initially fast for 5% and 10% vaccination rate thresholds, but became rather slow reaching the 30% vaccination level and above. The findings are robust to various applied estimation methods and model specifications.

**Conclusions:**

Democratic countries are much faster than authoritarian countries in their vaccination campaigns when controlling for other factors. This finding suggests that the quality of government and the political environment play a key role in popular support for government policies and programs. However, despite the early success of their vaccination campaigns, the democratic country group has been confronted with strong concerns of vaccine reluctance among their vast populations, indicating the two most potent variables explaining the speed of the COVID-19 vaccination campaign are education and economic conditions.

## Background

Vaccination is the dominant strategy to fight the Coronavirus Disease 2019 (COVID-19). Access to vaccines, the effectiveness of vaccination campaigns, and the acceptance among populations are determined by many factors like the political regimes, the state of the health system, the financial resources, and the educational conditions of the respective countries. The top priority is to reinstate normalcy after high numbers of deaths, overloaded sanitary systems, and huge economic burdens. Challenges are especially high for countries with incompetent health systems and hygiene facilities, as well as aging populations [4].

However, vaccine availability does not equate to vaccine accessibility [26]. In lower-income countries, even with the help of the World Health Organization through a vaccine alliance called COVID-19 Vaccines Global Access (COVAX), experts predict that only one out of ten people living in 70 poor income countries will be able to have access to COVID-19 vaccines by 2023 or later [13]. One reason is that '*Vaccine nationalism*' became popular amongst rich nations, thereby exacerbating the vaccine shortage [1, 5]. In contrast with low-income countries, by mid-August 2020, the richest countries (e.g., the United Kingdom, and the United States) have reserved enough doses of vaccines to inoculate each individual more than five times [6]. These wealthy countries also have secured the most extensive ranges and best vaccines [7].

Recent literature on COVID-19 suggests that democratic countries suffered more through higher infections but had lower case fatality rates [19]. While vaccine acceptance was highest in authoritarian countries (China, Vietnam), cash is used for vaccination promotion in some democratic countries like the United States, Serbia, Greece, and Canada [31]. Vaccination hesitancy of the population across policy regimes could lead to large differences in vaccination speed. Further, political-related anti-vaccine conspiracy theories that have been prevalent since the start of the COVID-19 pandemic are also strongly associated with reduced vaccination intentions [18]. Moore [23] also points out that conspiracy theorists are more prevalent amongst the poor and poorly educated.

Trust in science and confidence in vaccinations are strongly related. Social consensus on trust in science is substantially stronger in countries with higher levels of formal education. Studying data collected before the COVID-19 pandemic, Sturgis et al. [25] documented that people are more confident about vaccination in countries where trust in science is high. Based on large-scale, longitudinal, and representative surveys for 12 countries during the pandemic in 2020, also employing experimental data, Algan et al. [2] confirm that acceptance of non-pharmaceutical interventions, as well as vaccinations during COVID-19, are strongly related to perceived trust in scientists as the key driving force.

There is a large expectation that better education, in general, leads to a stronger vaccination acceptance rate among populations, although direct evidence in the economics literature is missing. For instance, Mayuko et al. [22] for the United States and Damiani et al. [11] for Italy found that the lower educated were less likely influenza vaccinated. Lu et al. [21] document that among adults aged 60 years or older, the better educated were more likely to receive herpes zoster vaccination than others. Khattak et al. [20] reveal that parents in Pakistan who had no education were more likely to refuse vaccination of their children. A major contribution of our study, therefore, is to provide extra knowledge on the effects of educational development on vaccination performance.

The effectiveness of a COVID-19 vaccination program is judged by how well it prevents the transmission of the virus. This can be separated into the evaluation of two steps: first, by studying the speed and the extend by how much a population gets vaccinated; and second, by measuring how strong the health effects after vaccinations are. The second stage is a complex modelling issue, where the necessary process to study is still not complete. Hence, we focus here on the first step and the initial process that is well documented. The study concentrates on two indicators of vaccination campaign performance, namely the vaccination rate and the pace at which a country scales up to meet a certain population vaccination rate. Because the adoption rate of the COVID-19 vaccine has been a significant obstacle for many countries, the sooner a country could increase its vaccination rate to protect more people, the more successful the vaccination program is.

Several elements, such as public education (which might lead to a greater vaccination acceptance rate), faith in government policy, vaccination access policy [14], investment to acquire vaccines, etc., could impede or accelerate the vaccination campaign speed. In addition, the larger the pressure from infections, the stronger the vaccination efforts of a country should be. To understand the factors affecting the scaling-up speed of the COVID-19 vaccination campaign better, this study reveals the relative strength of the effects of education, economic strength, political regimes, vaccination policies, and countries' specific demographic factors on how fast they implement and scale up vaccination campaigns in the context of diverse COVID-19 infection challenges.

The paper presents the used data and methodology in Data and methodology, followed by detailed regression analyses in Results, including weighted regressions, survival time analysis, and robustness checks. Discussion concludes.

## Data and methodology

For this study, country level data are used for 118 nations. Tables 7 and 8 in Appendix provide detailed explanations of variable definitions and data sources. The success of the COVID-19 vaccination campaign is investigated by studying the number of days countries needed since its respective start to reach certain vaccination shares among populations: Daysto1%/5%/10%/20%/30%. Choosing 30% as the upper bound ensures that our data set provides a reliable basis for studying the early vaccination success story across countries. Moving above would have caused a major loss of countries, with the potential of biased findings. Investigating the various thresholds from 1 to 30%, enables us to show the dynamic development of the effects of the interacting factors, in particular for the major determinants under investigation.

The major driving factors considered are political, educational, and economic backgrounds. The countries' classifications of political regimes include full democracy, flawed democracy, hybrid, and authoritarian regimes provided by the 2020 *Economist* democracy report (EIU, 2020) based on their democratic score calculated using key criteria such as electoral process and pluralism, functioning of government, political participation, political culture, and civil liberties. Compared to the well-known Freedom House index [15], the Economist Intelligence Unit Index (EIU Index) covers more democratic features such as political culture and participation. For the EIU Index, each of the 118 countries are allocated to one regime (1,0)-dummy for full democracy, flawed democracy, hybrid, and authoritarian (four variables). For the Freedom House Index, we have three (1,0)-dummy variables with values free, partial free and not free allocated in the same way. The sum of the dummy variables for both indices is 118. The expected years of schooling index [30] proxies the development of the educational system (YearsofSchooling), and the GDP per capita proxies the economic growth and capacity (GDPpercapita).

Other control variables are population density (PopulationDensity), population shares of ages 65 and older (Aged65older), vaccines purchased (VaccinesPurchased), vaccine access policies (VaccinePolicy), average daily new infected COVID-19 cases (January 2020 to October 11, 2021; AverageNewCases) to capture the relevance of societal pressures, and continent dummies. VaccinesPurchased (see Table 8 in Appendix) is the number of vaccines doses that countries have contracted to purchase from COVID-19 vaccine manufacturers according to Duke Global Health Innovation Center [13]. It controls for the government's commitment and efforts to get the vaccine available to the public, so international support efforts like COVAX are not included by intention. VaccinePolicy measures the level of access ability for the population from “0-no” to “5-universal” regulated by the government. The recorded number of new infected is influenced by various factors like definitions, availability of tests, testing behavior and government regulations and vary across countries, but it nevertheless captures the publicly perceived seriousness of the situation which drives government activity and the willingness of the population to follow recommendations and to adjust to the challenge. While the database reflects the situation until Fall 2021 only, the analysis is very relevant to contribute to the understanding of why and where the vaccination process observed got stuck thereafter.

Table 1 provides descriptive statistics of the variables used. According to Table 1, the average day for countries to reach 1% of vaccination level is approximately 36 days since the first case of vaccination. The days required to reach 5%, 10%, 20%, and 30% are about 73, 93, 116, and 134 days, on average, accordingly. Generally, vaccination campaigns globally speeded up as they moved up to higher uptake rates. The average GDP per capita for the sample is about 22,7 ($1,000). However, as the total number of countries included in the analysis is 118 countries, the GDP per capita widely varies from 0.92 to 116.9 ($1,000). Similarly, the YearsofSchooling variable measured in average expected school years also has a wide range of values, from 6.4 to 21.9 (years), with an average value of 14.1. The political regime variables (EUI Index, Freedom House Index) have means that represent the percentage of each category in the sample, and therefore the sum of the means add up to 1. For the EUI Index, 17.8% of the countries are allocated to full democracy, 37.3% to flawed democracy, 18.6% to hybrid regime, and 26.3% to authoritarian regime. For the Freedom House Index, 41.5% are considered as free, 33.1% as partial free, and 25.4% as not free.

**Table 1:** Descriptive statistics of vaccination speed and other variables for 118 countries studied (Obs. -number of countries, mean, standard deviation, and range of variable values)

Variable	Obs	Mean	Std. Dev	Min	Max
Daysto1%	118	35.95763	28.23328	0	129
Daysto5%	109	73.58716	44.02908	0	214
Daysto10%	104	93.69231	42.89071	4	222
Daysto20%	96	116.3333	40.39298	14	193
Daysto30%	91	134.5385	43.72752	24	229
AverageNewCases (to 1%)	118	67.7721	64.57344	0.0185814	254.0549
AverageNewCases (to 5%)	109	79.80594	69.7637	0.1911751	295.0323
AverageNewCases (to 10%)	104	87.68681	72.05924	0.4018115	340.575
AverageNewCases (to 20%)	96	100.0747	74.98198	0.6498013	361.3624
AverageNewCases (to 30%)	91	106.2103	77.77639	1.142303	356.5833
Full democracy	118	0.1779661	0.3841153	0	1
Flawed democracy	118	0.3728814	0.4856331	0	1
Hybrid regime	118	0.1864407	0.3911227	0	1
Authoritarian regime	118	0.2627119	0.441984	0	1
Free	118	0.4152542	0.4948672	0	1
Partial Free	118	0.3305085	0.4724022	0	1
Not Free	118	0.2542373	0.4372885	0	1
YearsofSchooling	118	14.257644	2.79901	6.47145	21.95433
GDPpercapita ($1,000)	118	22.76324	21.19165	0.926	116.9356
PopulationDensity (1,000 people/km2)	118	0.2758711	0.9824308	0.00198	7.915731
Aged65older (%)	118	10.09064	6.72996	1.144	27.049
VaccinePurchased (10 million units)	118	7.05846	27.32845	0	234.5
VaccinePolicy (1%)	118	1.797115	0.884347	0	4
VaccinePolicy (5%)	109	2.14452	0.7986983	0.6292135	4.664122
VaccinePolicy (10%)	104	2.34739	0.7963662	0.78	4.864865
VaccinePolicy (20%)	96	2.492691	0.7428048	0.7719298	4.864865
VaccinePolicy (30%)	91	2.638591	0.7204832	1	4.876543

*Notes:* Daysto 1/5/10/20/30%: number of days until 1%/5%/10%/20%/30% vaccination levels (total number of vaccine doses to total population); AverageNewCases: Average daily new infected COVID-19 cases per million of people since the first infected case recorded to the date of 1%/5%/10%/20%/30% vaccinated levels; YearsofSchooling: average number of years in formal education a person can expect to receive with the current enrolment rate at all levels of education; GDPpercapita: GDP per capita ($1000); PopulationDensity: population density index (1,000 people/km^2^); Aged65older: share of populations with age 65 and over (%); VaccinePurchased: number of vaccine doses contracted from COVID-19 vaccine manufacturers (10 million units); VaccinePolicy: average score of the COVID-19 vaccination policy chosen by countries overtime (0–5), the higher the score, the broader vaccination targets. Asia/Africa/Europe/Oceania/SouthAmerica: continent dummies

The main estimation method is weighted least squares (WLS) using population size as weights over ordinary least squares with robust standard errors, but we check for differences between the two methods in a separate robustness analysis. The advantage of WLS is efficiency, but ordinary least squares (OLS) with robust standard errors accounts for all types of heteroscedasticity ([32], p. 56). As usual, the regression is a linear combination Y = b X + e with Y an output variable, X a vector of independent variables, b the respective effect parameters including a constant, and e the error term. We also calculate the Owen–Shapley R^2^-decomposition [17] to judge the contributions of key variable groups. In addition, survival analysis using the Cox proportional hazards model [10] is also applied to explore the survival time distribution of reaching the 30% vaccination uptake rate, which serves as a failure event.

The dummy-variables for the political regimes cannot all be included, since they would be exactly collinear with the overall constant of the regression. The standard solution is to suppress one category and use it as the reference variable. Then the estimated coefficients measure the difference between the effect of the included category and the effect of the reference variable (see also Weighted regression analysis, first paragraph).

We did not include population size as an extra regressor in the analysis. Weighting by this variable when employing WLS covers for heteroscedasticity caused by this variable and weights all data points. Further, we include variants of country size through variables “GDPpercapita” and “PopulationDensity”, and have other regressors included depending on population size like “AverageNewCases” and “VaccinePurchased”.

## Results

### Weighted regression analysis

Table 2 shows the findings for vaccination levels 1%, 5%, 10%, 20%, and 30%, respectively, with rising R^2^'s from 0.556 for 1% to 0.730 for 30%. The numbers next to the variables are the estimated effects parameters with the standard errors below in parentheses indicating the precision of the estimates. *, **, and *** report the statistical significance at the 10%, 5% and 1% level, whereas *** suggests the highest reliability. For instance, the “-21.869* “ with “full democracy” in column 1 (Daysto1%) means that countries in that category reach the 1% vaccination level about 22 days faster than authoritarian countries (the left out reference category). The standard error of this coefficient is 11.448 with “*”, implying that the coefficient is statistically significant at the 10% level.

**Table 2:** Weighted least squares regression results for speed of COVID-19 vaccinations

	(1)	(2)	(3)	(4)	(5)
	Daysto1%	Daysto5%	Daysto10%	Daysto20%	Daysto30%
Democracy (reference group: authoritarian)					
Full	-21.869*	-18.624**	-21.015**	-12.673	-20.985
	(11.448)	(8.307)	(10.253)	(13.496)	(17.132)
Flawed	-19.927**	-23.632**	-15.869*	-2.844	0.571
	(8.799)	(9.930)	(9.234)	(10.824)	(12.519)
Hybrid	-17.589*	-30.058*	-35.121**	-19.774	-6.077
	(8.919)	(16.314)	(17.600)	(15.779)	(15.624)
YearsofSchooling	-5.444***	-7.946***	-7.382***	-6.859***	-3.949*
	(1.800)	(2.137)	(2.600)	(2.193)	(2.076)
AverageNewCases	-0.176***	-0.132**	-0.144***	-0.215***	-0.267***
	(0.065)	(0.059)	(0.054)	(0.074)	(0.080)
GDPpercapita	-0.152	-0.584*	-0.735**	-0.887***	-1.063***
	(0.196)	(0.304)	(0.296)	(0.293)	(0.301)
PopulationDensity	-1.622	0.141	-1.282	-0.816	-2.045
	(1.778)	(2.301)	(2.504)	(2.508)	(3.143)
Aged65older	0.697	1.122*	1.073	-0.351	-0.870
	(0.501)	(0.608)	(0.666)	(0.778)	(0.942)
VaccinePurchased	-0.004	-0.098***	-0.148***	-0.033	0.006
	(0.024)	(0.028)	(0.037)	(0.044)	(0.045)
VaccinePolicy	-5.691**	0.079	3.241	-3.948	-4.603
	(2.702)	(5.339)	(4.314)	(4.839)	(5.628)
Asia	-16.017*	-19.581**	-15.352*	-8.441	-4.375
	(8.419)	(7.836)	(8.217)	(9.161)	(10.659)
Africa	-3.605	25.273	18.766	-10.509	4.491
	(14.512)	(17.434)	(23.461)	(38.434)	(44.365)
Europe	-0.053	-18.168**	-16.788*	19.524	33.205**
	(7.684)	(7.635)	(8.648)	(11.823)	(14.080)
Oceania	19.805*	11.121	11.945	33.513*	32.039
	(11.823)	(15.993)	(17.448)	(18.360)	(20.200)
SouthAmerica	-0.047	-14.673	-19.106*	-8.486	-5.601
	(7.359)	(9.390)	(9.738)	(11.635)	(11.756)
Constant	157.727***	238.800***	250.561***	288.770***	277.442***
	(24.146)	(36.732)	(37.928)	(33.151)	(30.602)
*Number of countries*	118	109	104	96	91
Adj.*R*^*2*^	0.556	0.678	0.697	0.696	0.730
Owen–Shapley R-squared-decomposition (%R^2^):					
Political regimes	14.214	14.739	14.935	10.940	14.790
AverageNewCases	19.208	14.789	10.154	10.566	12.349
YearsofSchooling	24.086	19.079	15.110	18.740	17.775
GDPpercapita	10.568	11.880	17.699	34.495	34.728

*Notes:* Weighted least squares with population size. Daysto 1/5/10/20/30%: number of days until 1%/5%/10%/20%/30% vaccination levels (total number of vaccine doses to total population); AverageNewCases: Average daily new infected COVID-19 cases per million of people since the first infected case recorded to the date of 1%/5%/10%/20%/30% vaccinated levels; YearsofSchooling: average number of years in formal education a person can expect to receive with the current enrolment rate at all levels of education; GDPpercapita: GDP per capita ($1000); PopulationDensity: population density index (1,000 people/km^2^); Aged65older: share of populations with age 65 and over (%); VaccinePurchased: number of vaccine doses contracted from COVID-19 vaccine manufacturers (10 million units); VaccinePolicy: average score of the COVID-19 vaccination policy chosen by countries overtime (0–5), the higher the score, the broader vaccination targets. Asia/Africa/Europe/Oceania/SouthAmerica: continent dummies. Robust standard errors in parentheses: *p < 0.10, **p < 0.05, ***p < 0.01

The results reveal a negative association with democracy with authoritarian countries as the reference group. While estimated parameters are typically negative for the three types (full, flawed, and hybrid), significance and size vary. For thresholds 1%, 5%, and 10%, they are significant at least at the 10% level. 14.2% points of the *R*^2^ = 0.556 explanatory power in column (1) comes from these three political variables; political regimes explain 14.2% of the total variance of the Daysto1% total variance. This remains fairly stable in the vaccination process and is 14.8% points of *R*^2^ = 0.730 for threshold Daysto30%.

Educated countries also exhibit a negative and statistically significant effect on the duration of achieving vaccination thresholds. The percentage contributions of *YearsofSchooling* to the explained variation is 24.1% for Daysto1%, but are somewhat smaller but robust (19.1%, 15.1%, 18.7%, and 17.8%) in the next steps. Education is always more relevant for speeding up the process than political regimes.

The *GDPpercapita* effect is negative but small at the beginning and becomes large and strongly significant with rising vaccination thresholds. The Owen-Shapley decomposition allocates the highest contributions for Daysto10%, Daysto20% and Daysto30% with 17.7%, 34.5% and 34.7%, respectively. *GDPpercapita* also provides the strongest contribution among all variables, including the group of political regime variables from the 10% threshold level and so on.

*AverageNewCases* has throughout negative and 1% statistically significant coefficients. The pandemic provides extra incentives for countries to intensify the vaccination campaign. Measured by the Owen-Shapley contributions, this pressure is strongest for 1% and 5%. *VaccinePolicy* matters for Daysto1%, and *VaccinesPurchased* speeds up vaccinations for the 5% and 10% thresholds significantly. *PopulationDensity* and *Aged65older* are mostly not statistically significant. In comparison to North and Middle America, European countries vaccinated initially fast, similar to Asia (5% and 10% thresholds), but became rather slow in reaching the 30% vaccination level and above.

To summarize: When we study the evolution of the Owen–Shapley R-squared-decomposition at the bottom of Table 2 for our central variables, we can see some marked differences between the variables in the process: While the explanatory contribution is fairly stable below 15% for the set of political regime variables, education (YearsofSchooling) and infections (AverageNewCases) are very strong only at the beginning (for Daysto1% and Daysto5%), but then more relevant than Political regimes. Finally, economic strength (GDPpercapita) has the weakest contribution with 11–12% for Daysto1% and Daysto5%, but outperforms all other factors in particular for Daysto1% and Daysto5% with over 34%. Education becomes weaker in the process, but remains stronger than Political regimes and AverageNewCases. Hence, the detailed breakdown between vaccination levels from 1 to 30% provides valuable information.

### Survival time analysis

**Fig. 1:**
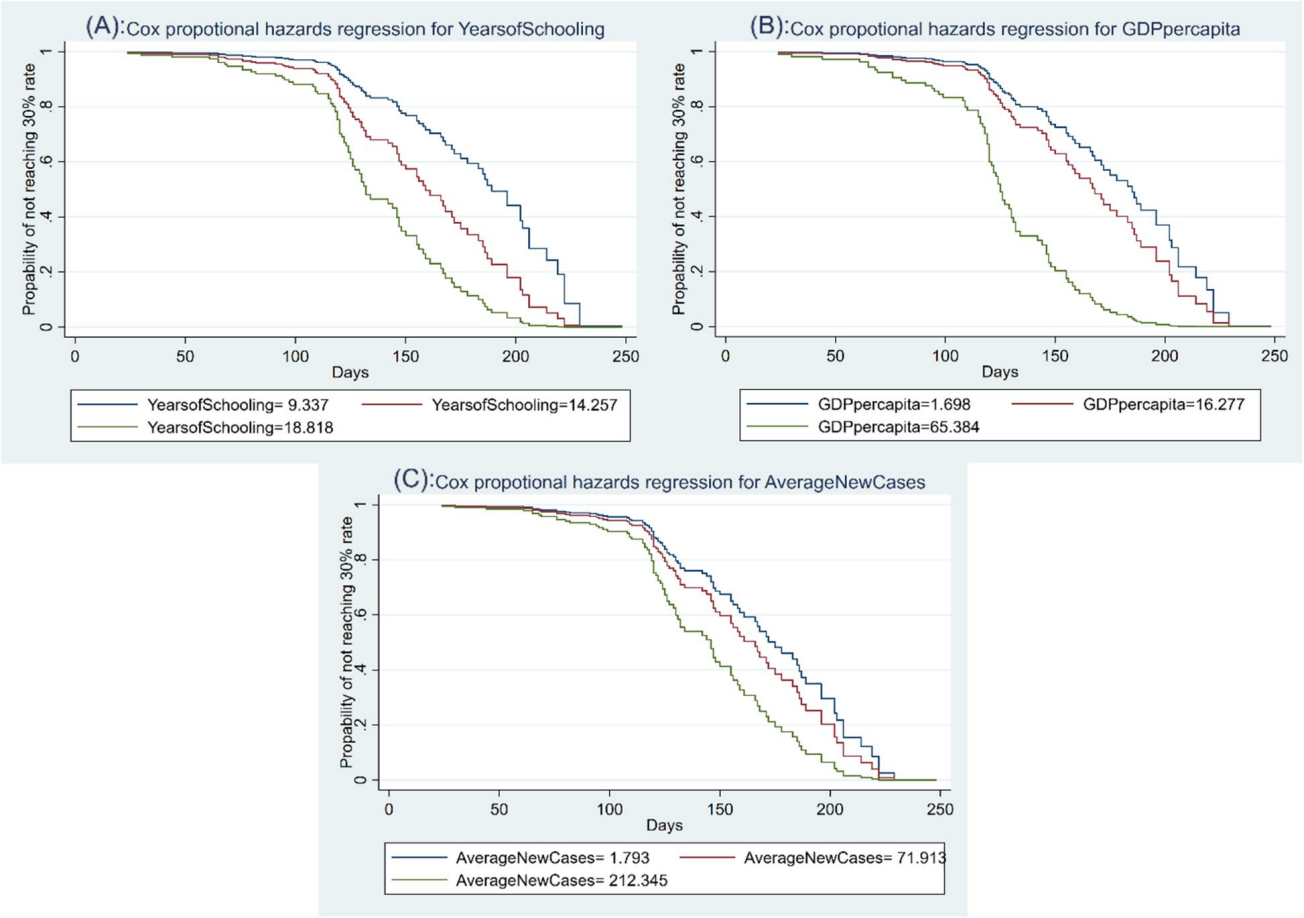
Predicted 250 days of reaching 30% vaccination uptake rate by YearsofSchooling, GDPpercapita, and AverageNewCases (at 5^th^, 50^th^ and 95^th^ percentiles values). Note: AverageNewCases: Average daily new infected COVID-19 cases per million of people since the first infected case recorded to the date of 1%/5%/10%/20%/30% vaccinated levels; YearsofSchooling: the average number of years in formal education that a person can expect to receive with the current enrolment rate at all level of education; GDPpercapita: GDP per capita ($1000). Panel **A**, **B**, **C** show that the probability to reach 30% vaccination uptake rate is higher (survival probability is smaller) for a country with higher values of YearsofSchooling, GDPpercapita and AverageNewCases at a given time under the analysis

Table 3 and Fig. 1 show the results of a survival time analysis for the event of reaching the 30% vaccination uptake rate using the Cox proportional hazards model [10]. The survival function shown in Fig. 1 gives the probability that a country will not reach the 30% uptake rate (survive the failure event) at a specific time t (number of days). Table 3 presents the estimated hazard ratios for the set of predictors used in Table 2. As a hazard ratio larger than 1 indicates a higher probability of the failure event, the results in Table 3 confirm that education levels (*YearsofSchooling*), Covid-19 new cases (*AverageNewCases*), and economic conditions (*GDPpercapita*) significantly increase the chance of reaching the 30% uptake rate for a country at a given time. (The estimates are significant at the 1% level). Democracy variables, however, are not significant in explaining the chances of reaching the 30% uptake rate. The results from the survival time analysis confirm the findings of the weighted regression analysis about the explanatory powers of a country's education levels, GDP, and infection situation for the speed of the vaccination campaign at its early stage.

**Table 3:** Cox proportional hazards model with failure event of reaching 30% vaccination uptake rate

	**Hazard Ratio**	**Standard Error**	**z-statistic**	**95% Confidence interval**	***p*-value**
Democracy (reference group: authoritarian):					
Full	1.440	0.745	0.71	(0.522–3.969)	0.481
Flawed	1.312	0.499	0.71	(0.621–2.768)	0.476
Hybrid	1.145	0.493	0.32	(0.492–2.666)	0.753
YearsofSchooling	1.162**	0.085	2.05	(1.006–1.343)	0.041
AverageNewCases	1.003**	0.001	2.02	(1.000–1.007)	0.043
GDPpercapita	1.025***	0.009	2.68	(1.006–1.044)	0.007
PopulationDensity	1.030	0.036	0.86	(0.961–1.105)	0.392
Aged65older	1.123	0.149	0.87	(0.865–1.458)	0.383
VaccinePurchased	1.000	0.003	0.11	(0.993–1.007)	0.909
Asia	1.237	0.503	0.52	(0.557–2.746)	0.6
Africa	0.149***	0.103	-2.74	(0.038–0.583)	0.006
Europe	0.527	0.258	-1.3	(0.201–1.379)	0.192
Oceania	0.552	0.429	-0.76	(0.120–2.536)	0.446
SouthAmerica	1.287	0.622	0.52	(0.498–3.323)	0.601

Test of proportional-hazards assumption: Chi-Square (χ^2^): 6.39; *p*-value: 0.956

Number of subjects (countries in analysis): 118

Number of failures (countries reach 30% vaccination uptake rate): 91

*Notes*: Breslow method for ties. AverageNewCases: Average daily new infected COVID-19 cases per million people since the first infected case recorded to the date of 1%/5%/10%/20%/30% vaccinated levels; YearofSchooling: the average number of years in formal education that a person can expect to receive with the current enrolment rate at all level of education; GDPpercapita: GDP per capita ($1000); PopulationDensity: population density index (1,000 people/km2); Aged65older: share of populations with age 65 and over (%); VaccinePurchased: number of vaccines doses which countries have contracted to purchase from COVID-19 vaccine manufacturers (10 million units); Asia/Africa/Europe/Oceania: dummy continent control. * *p* < 0.10, ** *p* < 0.05, *** *p* < 0.01

Figure 1 presents the distribution of survival times at 5^th^, 50^th^, and 95^th^ percentile values of *YearsofSchooling, GDPpercapita,* and *AverageNewCases*. The results suggest that after passing 100 days in vaccination campaigns, the probability of survival (not reaching 30% uptake rate) decreases substantially and much faster for the 95^th^ percentile compared to the 5^th^ and 50^th^ of all the three variables. For instance, at 150 days of the vaccination campaign, on average, the chance of not reaching the 30% uptake rate in a country that has an education level of about 18.8 years (95^th^ percentile) is only about 35%, compared to about 60% for a country with an average level of education (14 years) and about 80% for a country with 9.3 years of expected schooling (5^th^ percentile). This indicates that nations with high values for YearsofSchooling, GDPpercapita, and AverageNewCase are far more likely to attain an adoption rate of 30 percent much sooner than countries with lower values for these factors.

### Robustness results

Robustness tests are conducted to explore how sensitive our results are when different ways are employed to analyze the data.

First, we use the Freedom House democracy classifications (Free, Partial Free, Not Free) [16] instead of the regime metrics from the Economist democracy report (Table 2) to capture political features. Results in Table 4 suggest no conflicting findings in comparison to the results provided in Table 2. In general, a common observation is that democracy variables have significant impacts on the speed of the campaign at the very early stages of the vaccination campaign (1% and 5% uptake rates), but the differences fade away at higher uptake rate levels. The variance explanation power (Owen–Shapley R-squared) for political regimes variables is also very similar at around 14%. The effects of *YearsofSchooling*, *AverageNewCases,* and *GDPpercapita* remain strongly significant at the 1% level, and the size is very similar to what is observed in Table 2. Hence, all findings confirm the robustness of the results.

**Table 4:** Robustness tests (weighted least squares regressions) for speed of COVID-19 vaccinations with Freedom House democracy index for comparison with Table 2

	(1)	(2)	(3)	(4)	(5)
	Daysto1%	Daysto5%	Daysto10%	Daysto20%	Daysto30%
Democracy (Not Free is the base group):					
Free	-20.487	-14.548	-13.072	3.442	-3.734
	(14.787)	(10.487)	(13.118)	(16.944)	(18.826)
Partial Free	-15.284**	-29.659***	-11.445	-1.165	-0.118
	(6.643)	(11.225)	(13.510)	(12.519)	(12.790)
YearsofSchooling	-6.447***	-8.761***	-6.388***	-5.687***	-3.933**
	(1.679)	(1.791)	(2.160)	(1.712)	(1.643)
AverageNewCases	-0.163***	-0.192***	-0.170***	-0.235***	-0.214***
	(0.050)	(0.061)	(0.063)	(0.063)	(0.059)
GDPpercapita	-0.076	-0.572**	-0.618**	-0.909***	-1.178***
	(0.205)	(0.270)	(0.256)	(0.288)	(0.300)
PopulationDensity	-1.804	1.474	-3.124	-1.154	-1.026
	(2.005)	(2.925)	(3.778)	(3.433)	(3.948)
Aged65older	0.945	0.846	0.862	-1.108	-1.504
	(0.849)	(0.930)	(0.951)	(1.189)	(1.329)
VaccinePurchased	-0.007	-0.073***	-0.116***	-0.000	0.017
	(0.018)	(0.019)	(0.029)	(0.024)	(0.024)
VaccinePolicy	-3.241	2.257	6.062	-4.952	-5.969
	(3.303)	(4.807)	(4.678)	(5.060)	(5.982)
Asia	-19.029**	-26.848***	-19.752*	-9.758	-6.454
	(8.958)	(10.175)	(11.552)	(10.884)	(12.142)
Africa	-3.425	15.263	21.918	-13.260	3.075
	(13.073)	(21.405)	(29.764)	(43.730)	(48.036)
Europe	-1.265	-16.693*	-16.201*	24.215*	33.108**
	(8.609)	(8.851)	(9.378)	(12.799)	(14.388)
Oceania	21.954**	7.764	-3.099	20.730	26.961
	(10.438)	(14.902)	(13.545)	(14.955)	(16.907)
SouthAmerica	0.324	-17.001	-16.721	-7.510	-8.243
	(8.211)	(10.629)	(10.176)	(11.842)	(11.559)
Constant	161.860***	252.907***	225.113***	275.490***	283.982***
	(24.395)	(37.276)	(34.474)	(33.264)	(31.316)
*Number of countries*	118	109	104	96	91
Adj. *R*2	0.551	0.694	0.666	0.680	0.719
Owen–Shapley R-squared decomposition (% R2):					
Political regimes	14.129	13.797	11.099	10.600	15.123
AverageNewCases	19.313	14.520	10.288	9.828	10.718
YearOfSchooling	23.046	19.271	15.034	18.947	18.088
GDPpercapita	9.202	11.130	16.072	34.036	35.109

*Notes:* Weighted least squares with population size. Daysto 1/5/10/20/30%: number of days until the 1%/5%/10%/20%/30% COVID-19 vaccination levels (total number of vaccine doses to total population); AverageNewCases: Average daily new infected COVID-19 cases per million of people since the first infected case recorded to the date of 1%/5%/10%/20%/30% vaccinated levels; YearsofSchooling: the average number of years in formal education that a person can expect to receive with the current enrolment rate at all level of education; GDPpercapita: GDP per capita ($1000); PopulationDensity: population density index (1,000 people/km2); Aged65older: share of populations with age 65 and over (%); VaccinePurchased: number of vaccines doses which countries have contracted to purchase from COVID-19 vaccine manufacturers (10 million units); VaccinePolicy: average score of the COVID-19 vaccination policy chosen by a countries overtime (0–5), the higher the score, the broader vaccination targets are. Asia/Africa/Europe/Oceania: dummy continent control. Robust standard errors are in parentheses. * *p* < 0.10, ** *p* < 0.05, *** *p* < 0.01

Second, we use standard OLS regression instead of WLS regression for estimation with robust standard errors to deal with general forms of heteroscedasticity. OLS treats all states alike, whereas WLS with population weighting delivers people-weighted results. A proper choice might not be obvious, but large differences in the findings would be problematic. Table 5 shows that for our data the core story remains the same, which is a standard finding in the literature comparing OLS with WLS (see [3] pp. 202–203, for instance). Political regime variables show weak impacts while remaining negatively associated with the days required. *YearsofSchooling, AverageNewCases,* and *GDPpercapita* still show substantial effects at either 1% or 10% significance levels even though they are generally lower than for the WLS regressions. However, the R^2 −^ measures in all five columns in Table 5 (from 17 to 38%) are much smaller than those in Table 2 (from 55 to 73%) using the same set of explanatory variables.

**Table 5:** Standard OLS regression results for speed of COVID-19 vaccinations for comparison with Table 2

	(1)	(2)	(3)	(4)	(5)
	Daysto1%	Daysto5%	Daysto10%	Daysto20%	Daysto30%
Democracy (Authoritarian is the base group):					
Full	-4.009	-7.786	-12.665	-3.682	-7.794
	(9.763)	(13.368)	(13.427)	(16.260)	(18.225)
Flawed	-10.284	-16.398	-18.784*	-8.194	-7.502
	(8.885)	(11.794)	(11.054)	(14.526)	(16.051)
Hybrid	-7.632	-15.165	-21.218	-9.167	2.172
	(9.865)	(15.311)	(15.058)	(17.614)	(16.323)
YearsofSchooling	-4.265***	-5.838***	-4.322*	-3.509	-3.410
	(1.586)	(2.148)	(2.244)	(2.340)	(2.377)
AverageNewCases	-0.088***	-0.118***	-0.077	-0.077	-0.116*
	(0.032)	(0.042)	(0.047)	(0.053)	(0.062)
GDPpercapita	-0.104	-0.322	-0.533**	-0.643***	-0.666**
	(0.152)	(0.201)	(0.207)	(0.241)	(0.266)
PopulationDensity	-1.564	-2.396	-1.597	-1.592	-1.721
	(1.753)	(2.769)	(2.985)	(3.182)	(3.758)
Aged65older	0.440	0.464	0.780	-0.197	-0.451
	(0.551)	(0.927)	(0.930)	(1.136)	(1.402)
VaccinePurchased	0.025	-0.001	-0.026	0.031	0.110
	(0.029)	(0.052)	(0.055)	(0.085)	(0.089)
VaccinePolicy	-0.894	1.658	6.215	0.530	-0.624
	(3.102)	(5.326)	(5.087)	(6.677)	(8.432)
Asia	-4.805	-2.791	-11.421	-6.463	-2.858
	(8.362)	(11.104)	(11.625)	(14.465)	(16.656)
Africa	7.083	28.614	22.964	8.783	9.671
	(11.632)	(17.610)	(17.960)	(23.235)	(33.506)
Europe	1.644	3.517	-8.848	6.938	24.548
	(8.019)	(12.670)	(13.138)	(16.084)	(18.842)
Oceania	13.289	-15.239	-24.625	-16.433	-10.125
	(21.171)	(31.686)	(29.959)	(34.243)	(44.277)
SouthAmerica	8.818	5.637	-5.206	-3.966	1.139
	(9.370)	(11.472)	(12.540)	(16.213)	(17.484)
Constant	106.247***	173.544***	173.142***	200.492***	219.318***
	(22.559)	(34.997)	(32.932)	(37.935)	(37.462)
*Number of countries*	118	109	104	96	91
Adj. *R*2	0.283	0.383	0.388	0.172	0.175

*Notes:* Daysto 1/5/10/20/30%: number of days until the 1%/5%/10%/20%/30% COVID-19 vaccination levels (total number of vaccine doses to total population); AverageNewCases: Average daily new infected COVID-19 cases per million of people since the first infected case recorded to the date of 1%/5%/10%/20%/30% vaccinated levels; YearsofSchooling: the average number of years in formal education that a person can expect to receive with the current enrolment rate at all level of education; GDPpercapita: GDP per capita ($1000); PopulationDensity: population density index (1,000 people/km2); Aged65older: share of populations with age 65 and over (%); VaccinePurchased: number of vaccines doses which countries have contracted to purchase from COVID-19 vaccine manufacturers (10 million units); VaccinePolicy: average score of the COVID-19 vaccination policy chosen by a countries overtime (0–5), the higher the score, the broader vaccination targets are. Asia/Africa/Europe/Oceania: dummy continent control. Robust standard errors are in parentheses. * *p* < 0.10, ** *p* < 0.05, *** *p* < 0.01

Third, taking variables *AverageNewCases*, *VaccinesPurchased,* and *VaccinePolicy* out of the regressions leads to more substantial and all significant effects for *GDPpercapita,* and to more negative and more significant estimates for the *Political Regime* variables. Still, the qualitative story remains the same (Table 6). *YearsofSchooling* shows somewhat weaker and less significant effects. Obviously, those factors are also associated with the pandemic and vaccine-related control variables.

**Table 6:** Weighted least squares regression results for speed of COVID-19 vaccination programs (without pandemic and vaccine-related control variables for comparison with Table 2)

	(1)	(2)	(3)	(4)	(5)
	Daysto1%	Daysto5%	Daysto10%	Daysto20%	Daysto30%
Democracy (Authoritarian is the base group):					
Full	-18.858*	-20.372*	-24.861**	-11.532	-15.092
	(10.331)	(10.671)	(12.274)	(15.041)	(18.637)
Flawed	-24.279***	-38.909***	-39.235***	-18.208	-13.563
	(8.015)	(9.922)	(10.429)	(11.118)	(14.210)
Hybrid	-17.810*	-30.312**	-35.158*	-25.636*	-18.920
	(9.381)	(15.084)	(19.223)	(15.203)	(16.575)
YearsofSchooling	-4.885**	-6.714***	-5.620	-6.075**	-4.478*
	(1.873)	(2.396)	(3.471)	(2.567)	(2.305)
GDPpercapita	-0.534**	-0.854***	-1.049***	-1.335***	-1.620***
	(0.213)	(0.298)	(0.325)	(0.297)	(0.312)
PopulationDensity	0.547	0.052	-1.432	0.713	1.623
	(2.435)	(3.669)	(5.765)	(4.504)	(4.748)
Aged65older	1.041*	1.044	0.780	-0.168	-0.614
	(0.550)	(0.685)	(0.840)	(0.888)	(1.035)
Asia	-10.400	-13.102	-7.109	-2.420	0.730
	(9.869)	(11.514)	(12.826)	(11.717)	(12.424)
Africa	0.627	35.592*	31.758	-6.658	5.652
	(15.184)	(19.997)	(25.209)	(38.172)	(40.703)
Europe	-8.365	-14.905	-8.125	9.215	17.116
	(9.125)	(10.114)	(10.572)	(11.191)	(12.652)
Oceania	28.158**	19.618	24.757	41.887**	48.170**
	(12.664)	(16.729)	(20.495)	(18.299)	(18.397)
SouthAmerica	-10.884	-11.432	-11.191	-18.550	-23.561*
	(11.569)	(12.851)	(12.262)	(13.103)	(12.902)
Constant	134.481***	216.246***	228.183***	264.914***	270.952***
	(24.583)	(34.889)	(47.471)	(36.488)	(34.319)
*Number of countries*	118	109	104	96	91
Adj. *R*2	0.513	0.635	0.603	0.647	0.691

*Notes:* Weighted least squares with population size as weight. Daysto 1/5/10/20/30%: number of days until the 1%/5%/10%/20%/30% COVID-19 vaccination levels (total number of vaccine doses to total population); YearsofSchooling: the average number of years in formal education that a person can expect to receive with the current enrolment rate at all level of education; GDPpercapita: GDP per capita ($1000); PopulationDensity: population density index (1,000 people/km2); Aged65older: share of populations with age 65 and over (%). Asia/Africa/Europe/Oceania: dummy continent control. Robust standard errors are in parentheses. * *p* < 0.10, ** *p* < 0.05, *** *p* < 0.01

**Table 7:** Full names and associated Alpha-3 codes of 118 countries

Countries	Codes	Countries	Codes	Countries	Codes
Afghanistan^b^	AFG	Hungary	HUN	Panama	PAN
Angola^c^	AGO	India	IND	Papua New Guinea^a^	PNG
Argentina	ARG	Indonesia	IDN	Paraguay	PRY
Australia	AUS	Iraq^c^	IRQ	Peru	PER
Austria	AUT	Ireland	IRL	Philippines	PHL
Bahrain	BHR	Israel	ISR	Poland	POL
Belarus	BLR	Italy	ITA	Portugal	PRT
Belgium	BEL	Jamaica^d^	JAM	Qatar	QAT
Benin^a^	BEN	Japan	JPN	Romania	ROU
Bolivia	BOL	Jordan	JOR	Russia	RUS
Bosnia and Herzegovina	BIH	Kazakhstan	KAZ	Rwanda^4^	RWA
Botswana^d^	BWA	Kenya^b^	KEN	Saudi Arabia	SAU
Brazil	BRA	Kuwait	KWT	Senegal^c^	SEN
Bulgaria	BGR	Kyrgyzstan^d^	KGZ	Serbia	SRB
Burkina Faso^a^	BFA	Laos	LAO	Singapore	SGP
Cambodia	KHM	Latvia	LVA	Slovakia	SVK
Cameroon^a^	CMR	Lebanon	LBN	Slovenia	SVN
Canada	CAN	Libya^d^	LBY	South Africa	ZAF
Chile	CHL	Lithuania	LTU	South Korea	KOR
Colombia	COL	Luxembourg	LUX	Spain	ESP
Costa Rica	CRI	Malaysia	MYS	Sri Lanka	LKA
Croatia	HRV	Mali^a^	MLI	Sweden	SWE
Czechia	CZE	Malta	MLT	Switzerland	CHE
Denmark	DNK	Mauritius	MUS	Tajikistan	TJK
Dominican Republic	DOM	Mexico	MEX	Thailand	THA
Ecuador	ECU	Moldova	MDA	Togo^c^	TGO
Egypt^c^	EGY	Mongolia	MNG	Trinidad and Tobago	TTO
El Salvador	SLV	Morocco	MAR	Turkey	TUR
Estonia	EST	Mozambique^c^	MOZ	Uganda^b^	UGA
Fiji	FJI	Namibia^c^	NAM	Ukraine	UKR
Finland	FIN	Nepal	NPL	United Arab Emirates	ARE
France	FRA	Netherlands	NLD	United Kingdom	GBR
Gabon^b^	GAB	New Zealand	NZL	United States	USA
Georgia	GEO	Nicaragua^c^	NIC	Uruguay	URY
Germany	DEU	Niger^a^	NER	Venezuela	VEN
Ghana^b^	GHA	Nigeria^a^	NGA	Vietnam	VNM
Greece	GRC	Norway	NOR	Yemen^a^	YEM
Guatemala	GTM	Oman	OMN	Zambia^a^	ZMB
Honduras	HND	Pakistan	PAK	Zimbabwe	ZWE
Hong Kong	HKG				

*Note*: This table describes the full names and codes of 118 countries in model (1) in Table 2

^a^ Countries that are not available for analyses in models (2), (3), (4), and (5) because they have not reached the vaccination rates of 5%, 10%, 20%, and 30%

^b^ Countries that are not available for analyses in models (3), (4), and (5) because they have not reached the vaccination rates of 10%, 20%, and 30%

^c^ Countries that are not available for analyses in models (4), and (5) because they have not reached the vaccination rates of 20%, and 30%

^d^ Countries that are not available for analyses in model (5) because they have not reached the vaccination rates of 30%

**Table 8:** List of variables used, definitions and data sources

Variables	Description	Sources
Daysto1%	Number of days needed to achieve the 1%/5%/10%/20%/30% COVID-19 vaccination levels (total number of doses to total population)	Ritchie et al. [24]
Daysto5%		
Daysto10%		
Daysto20%		
Daysto30%		
YearsofSchooling	The average number of years in formal education that a person can expect to receive with the current countries' enrolment rates at all levels of education	UNDP [30]
Democracy classifications according to countries' democracy index score from The Economist	***Full democracy:*** all areas such as basic political freedoms, civil liberties, political culture, government functioning are at a high level	The Economist Intelligence Unit [29]
	***Flawed democracy:*** free and fair elections, basic civil liberties respected but suffered from problems in government functioning, underdeveloped political cultures, and weak political participant	
	***Hybrid regime:*** substantial irregularities in election, more prevalent problems in government functioning, underdeveloped political cultures, the rule of law is weak, and corruption is widespread, no independent judiciary	
	***Authoritarian:*** state political plurals are absent, outright dictatorship, elections are not free and fair and infringements of civil liberties, no independent judiciary	
Democracy classifications according to countries' democracy index score from The FreedomHouse	The democracy index score is based on the aggregate score of two main dimensions: Political rights (0–40) and Civil liberties (0–60). The total Political Rights and Civil Liberties scores are equally weighted in this calculation and based on those classifications (***Free/Partial Free/Not Free***) are decided	Freedom House [16]. 'Freedom in the world 2021'
AverageNewCases (1%/5%/10%/20%/30%)	Average daily new infected COVID-19 cases per million to the date of 1%/5%/10%/20%/30% vaccinated levels	Ritchie et al. [24]
PopulationDensity (1,000 people/km2)	Population density index	World Bank national accounts data
Aged65older (%)	Share of populations with age 65 and over	World Bank national accounts data
GDPpercapita ($1,000)	Gross domestic product per capita	World Bank national accounts data
VaccinePurchased (10 million units)	Number of vaccines doses that countries have contracted to purchase from COVID-19 vaccine manufacturers	Duke Global Health Innovation Center [13]
VaccinePolicy (1%/5%/10%/20%/30%)	Countries are grouped into six categories: 0: No availability 1: Availability for ONE of following: key workers/ clinically vulnerable groups / elderly groups 2: Availability for TWO of following: key workers/ clinically vulnerable groups / elderly groups 3: Availability for ALL of following: key workers/ clinically vulnerable groups / elderly groups 4: Availability for all three plus partial additional availability (select broad groups/ages) 5: Universal availability Average score of Vaccine policies calculated based on the changes of vaccine policies since the beginning of the vaccine program to the 1%/5%/10%/20%/30% vaccination level (total doses/total population)	Ritchie et al. [24]

*Note:* This table shows the description and sources of variables used in this study

## Discussion

Prior research has focused mostly on behavioral sciences to increase vaccination rates [28]. A study from Japan demonstrates that the message of getting vaccinated may encourage others to become immunized [27]. The 'nudges' increased the proportion of older individuals who would take a free vaccine if offered but did not seem to affect younger adults [27]. Chang et al. [9] demonstrated that in the United States, neither financial incentives, diverse public health messages, nor a simple vaccination appointment planner could increase vaccination rates among vaccine-hesitant individuals. Improving adoption would request more stringent regulatory levers, ranging from workplace standards to government regulations. In contrast, [8] found that even a little financial incentive of €20 dramatically increases immunization rates. In the face of these inconsistent findings, our study provides empirical evidence on country-level data from 118 countries and gives worldwide evidence about the critical role of political factors, education, economic conditions, and infection rates in the pace of vaccination campaigns.

In December 2020, democratic countries like the United Kingdom, Germany, and the United States were among the few first nations to launch their COVID-19 vaccination program. Our results suggest that the democratic country group was much faster than the authoritarian group in their vaccination campaign when controlling for other factors. This finding is consistent with the studies of Davidovic and Harring [12] and Youde [33], which suggest that the quality of government and political environment has a crucial impact on popular support for government policies and programs. However, despite the early success of their vaccination campaigns, these nations have been confronted with strong concerns of vaccine reluctance among their vast populations, indicating the critical roles of factors like education and economic conditions. Notably, countries with higher education levels tend to be faster in their vaccination pace to protect their people. This result is consistent with past studies confirming, in general, that higher levels of education increase the vaccine acceptability rate among the public [, 11, 22].

### Limitations

Beyond all the valuable information, this study provides, there are also a number of limitations and suggestions for future research. The data used provided only valuable information for the early stages of the vaccination process which started diverse across the countries of the world for various reasons. Our major global factors worked quite differently during the different levels of vaccination success up to 30%. We found too few countries available above that level to study higher rates of success with sufficient credibility. It may well be that for instance the role of political regimes provides a quite different pattern, in particular when it comes to vaccine resistance. This also suggests that future work needs to study this process together with a more differentiated view on the number of repeated vaccinations, different degrees of vaccination resistance and the different variants of the Covid-19 challenge.

A completely different angle is the study of the effectiveness of a COVID-19 vaccination program by evaluating how well it prevents the transmission of the virus. Data for this only slowly gets available, and this interacts also in a complex way with the many other incentives and policies affecting individual behavior. This requests not only good data but also complex modelling.

Further, this study used only country-wide data to provide empirical evidence globally. It is one of the strengths but also a weakness when this setup cannot explain the factors affecting the success of vaccination campaigns through different periods of time. It covers the dynamics of the vaccination process by comparing country data from the point of departure of the availability of vaccines in the countries. But this neither enables us to study diverse government strategies over time, nor can we make use of individual data. Panel data at the national level and the combination with individual, in particular longitudinal data could be promising when we have much longer periods of experience with vaccinations at hand. Finally, measurement problems, partly because of missing standardizations, partly because of missing observations, may cause biases which need further reflections how the robustness of the findings of this study can be examined.

## Conclusions

As vaccination is the key to fight COVID-19 so far, this study aims to explore factors that influence the speed of a COVID-19 vaccination campaign at its early stages (from 1 to 30% uptake rates). Based on country-level data from 118 countries globally, this research provides empirical evidence on the impacts of countries' political regimes, education levels, economic conditions, COVID-19 infection situation, and other factors (vaccination policies or vaccination purchased) on vaccination campaign progression. Using the weighted least squared and survival analysis, it is found that the two most potent variables explaining the speed of the COVID-19 vaccination campaign are countries' education level and GDP per capita. More democratic countries only have an initial advantage, but the effects fade away as vaccination campaigns progress to higher levels of uptake rates. The intensities of COVID-19 new infected cases in a country also support early vaccination speed. Additionally, compared to the North and Middle American countries, European and Asia countries are pretty fast initially (from 1 to 10% uptake rates) but become slower in reaching higher vaccination levels (at 30% uptake rate).

## Data Availability

The data is publicly available as documented in the Appendix.

## Appendix

Tables 7 and 8

## Abbreviations

COVID-19: Coronavirus Disease 2019
COVAX: COVID-19 Vaccines Global Access
EIU: Economist Intelligence Unit
GDP: Gross Domestic Product
OLS: Ordinary Least Squared Regression
WLS: Weighted Least Squares
